# C-terminal processing of yeast Spt7 occurs in the absence of functional SAGA complex

**DOI:** 10.1186/1471-2091-8-16

**Published:** 2007-08-08

**Authors:** Stephen MT Hoke, Gaoyang Liang, A Irina Mutiu, Julie Genereaux, Christopher J Brandl

**Affiliations:** 1 Department of Biochemistry, Schulich School of Medicine & Dentistry, University of Western Ontario, London, N6A5C1, Canada; 2 Department of Biochemistry and Biophysics, University of North Carolina at Chapel Hill, 27599-7295, USA

## Abstract

**Background:**

Spt7 is an integral component of the multi-subunit SAGA complex that is required for the expression of ~10% of yeast genes. Two forms of Spt7 have been identified, the second of which is truncated at its C-terminus and found in the SAGA-like (SLIK) complex.

**Results:**

We have found that C-terminal processing of Spt7 to its SLIK form (Spt7_SLIK_) and to a distinct third form (Spt7_Form3_) occurs in the absence of the SAGA complex components Gcn5, Spt8, Ada1 and Spt20, the latter two of which are required for the integrity of the complex. In addition, N-terminally truncated derivatives of Spt7, including a derivative lacking the histone fold, are processed, indicating that the C-terminus of Spt7 is sufficient for processing and that processing does not require functional Spt7. Using galactose inducible Spt7 expression, we show that the three forms of Spt7 appear and disappear at approximately the same rate with full-length Spt7 not being chased into Spt7_SLIK _or Spt7_Form3_. Interestingly, reduced levels of Spt7_SLIK _and Spt7_Form3 _were observed in a strain lacking the SAGA component Ubp8, suggesting a regulatory role for Ubp8 in the truncation of Spt7.

**Conclusion:**

We conclude that truncation of Spt7 occurs early in the biosynthesis of distinct Spt7 containing complexes rather than being a dynamic process linked to the action of the SAGA complex in transcriptional regulation.

## Background

The multisubunit SAGA (Spt-Ada-Gcn5-Acetyltransferase) complex is a prototype for transcriptional coactivators that interface between DNA binding transcriptional regulators, the chromosomal template and the basal transcriptional machinery [1]. SAGA is a dynamic entity, with components existing in subcomplexes having related but distinct functions [2-6].

The Ada components of SAGA (*ADA2, NGG1/ADA3, GCN5/ADA4*) were first identified based on their involvement in regulated transcription in *Saccharomyces cerevisiae *[7-11]. SAGA possesses histone acetyltransferase (HAT) activity mediated by Gcn5 [2,12], which stimulates or represses transcription in a promoter specific fashion [13-17]. The structural core of SAGA is composed of a subset of the TBP-associated factors (TAFs) that are also found in TFIID [18,19]. Spt7, Ada1 and Spt20, members of the TBP-subclass of Spt (Suppressors of Ty insertions) [2,20-22] proteins, are located near the core of the complex and are required for its integrity, forming an interface between TAFs and other SAGA-specific components [19]. The additional Spt proteins, Spt3 and Spt8 are involved in direct interactions with TBP [3,22-25]. Tra1, a 437 kDa, phosphatidylinositol-3-kinase related protein [26,27] is found at the periphery of the complex and interacts with DNA-binding transcriptional regulators thus conferring promoter specificity to SAGA [28-32]. Through mass spectrometry analysis of purified SAGA complex, other components have been identified [33-36]. One of these proteins, Sus1, is associated with the nuclear pore mRNA export machinery [35] suggesting that SAGA may have a broader role in nuclear processes than previously envisioned.

Two forms of Spt7 have been described. The full-length protein of 1332 amino acid residues is found within the SAGA complex [2]. The second, a C-terminally truncated form likely arising from processing between residues 1125 and 1150 is found in the SLIK (SAGA-like) complex [3-6]. As suggested by the alternate name for this complex, SALSA (SAGA altered, Spt8 absent), SLIK also differs from SAGA in that it lacks Spt8 [3,4]. The absence of Spt8 can be attributed to the fact that it interacts with Spt7 in the region deleted in the truncated form [4,6]. The truncation of Spt7 and the subsequent loss of Spt8 may be a mechanism to create a complex that acts without effects mediated through TBP; for example, the derepression of the *HIS3 *promoter under starvation conditions [3,4]. A further difference between SAGA and SLIK is that Rtg2, which is required for the retrograde response pathway in yeast [37], is found specifically in SLIK [5]. Two other Rtg proteins, Rtg1 and Rtg3 are transcription factors required for the expression of retrograde target genes such as *CIT2 *[38]. Pray-Grant et al. [5] propose that SLIK facilitates transcriptional activation by Rtg1 and Rtg3 and perhaps also at a distinct set of promoters. However, the exact role of SLIK is unclear as the interpretation of transcriptional effects upon disruption of *RTG2 *is complicated since Rtg2 is required for nuclear import of Rtg1 and Rtg3 [39].

Two models for the relationship between the Spt7-containing complexes can be envisioned. In the first, processing at the C-terminus would occur as part of the biosynthesis of distinct SAGA and SLIK complexes. Alternatively, processing might occur during the normal functioning of SAGA, perhaps to irreversibly signal that a transcriptional event had occurred and/or enabling subsequent activity of the SLIK complex. In this study we performed experiments to differentiate between these models. We show that the conversion of Spt7 from its full-length SAGA-form (Spt7_SAGA_) to its truncated SLIK-form (Spt7_SLIK_) and a third C-terminally processed form (Spt7_Form3_) occurs in the absence of components of the complex including Ada1 and Spt20, which are required for the integrity of the complex. Processing to these forms occurs rapidly after its synthesis and in the absence of full-length Spt7. Taken together our results suggest that processing of Spt7 occurs in the biosynthesis of distinct Spt7-containing complexes.

## Results and discussion

### Truncated forms of Spt7 occur in the absence of fully functional SAGA complex

**Figure 1:**
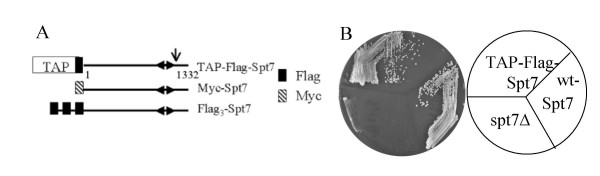
*Epitope-tagged Spt7 molecules used in this study*. **A **TAP-Flag-Spt7 contains N-terminal TAP and Flag tags fused in frame to the initiator Met of Spt7 (1332 amino acids) via a triple alanine linker encoded by a *NotI *site. Myc-Spt7 has a similar constructed N-terminal myc-epitope while Flag_3_-Spt7 contains 3 tandem copies of the Flag epitope. The position of the histone fold (amino acids 979–1045; [43]) is indicated with inverted arrows. An arrow indicates the approximate position of the cleavage site that generates Spt7_SLIK_. **B**. *TAP-Flag-Spt7 is functional*. Yeast strains BY4741 (*SPT7*), BY3218 (*spt7Δ0*) and BY3218 transformed with *YCpDed-TAP-Flag-SPT7 *were grown on a minimal plate depleted of inositol for 3 days at 30°C.

Two forms of Spt7 have been described that differ in their length [3-6]. Full-length Spt7 is 1332 amino acid residues and is found in the SAGA complex. This form, Spt7_SAGA_, migrates with an apparent molecular mass of approximately 200 kDa on SDS-PAGE. The SLIK complex contains a C-terminal truncation of Spt7 that results in an apparent molecular mass of approximately 180 kDa [4]. Based upon the mobility of this form and the differential function of Spt7 derivatives with C-terminal deletions, Spt7_SLIK _is predicted to contain a C-terminal truncation of approximately 200 amino acids [4,6]. To detect these forms of Spt7 we engineered a centromeric plasmid expressing a TAP and Flag-tagged version of Spt7 that could be identified after C-terminal truncation (*YCpDed-TAP-Flag-SPT7*, Figure 1a). We first addressed whether this molecule was functional by determining if it would complement a deletion of *spt7 *as assayed by growth on media depleted for inositol. As shown in Figure 1b, a strain lacking *SPT7 *is unable to grow on media lacking inositol. *YCpDed-TAP-Flag-SPT7 *allows for near complete restoration of growth.

**Figure 2:**
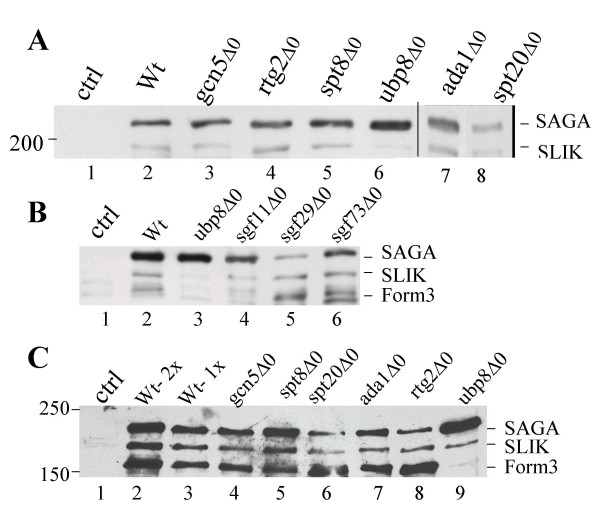
*Processed forms of Spt7 in yeast deletion strains*. **A**. Wild-type (BY4741) or the indicated yeast deletion strains (*gcn5Δ0*, *rtg2Δ0*, *spt8Δ0*, *ubp8Δ0*, *ada1Δ0*, *spt20Δ0*; lanes 3–8, respectively) were transformed with YCpDed-TAP-Flag-*SPT7*, with the exception of lane 1 (ctrl) which was transformed with the control vector YCp-TAP-Flag. Cells were grown to an A_600 _= 1.0–1.5 in YP containing 2% glucose and crude cell lysates prepared by glass bead disruption. 50 μg of protein was separated by 5% SDS-PAGE and Western blotted with anti-Flag antibody. The mobility of a 200 kDa molecular weight marker is indicated on the left. Spt7_SAGA _and Spt7_SLIK _are marked. **B**. BY4741 (Wt, lane 2) and the indicated deletion strains (*ubp8Δ0*, lane 3; *sgf11Δ0*, lane 4; *sgf29Δ0*, lane 5; *sgf73Δ0*, lane 6) containing TAP-Flag-Spt7 were grown as above, crude yeast extracts separated by SDS-PAGE and Western blotted with anti-Flag-antibody. Lane one contains crude extract from the wild-type strain BY4741. Spt7_SAGA_, Spt7_SLIK _and Spt7_Form3 _are marked. **C**. Wild-type (BY4741 lanes 2 and 3) or the indicated yeast deletion strains, *gcn5Δ0 *(lane 4), *spt8Δ0 *(lane 5), *spt20Δ0 *(lane 6), *ada1Δ0 *(lane 7), *rtg2Δ0 *(lane 8), *ubp8Δ0 *(lane 9) containing YCpDed-TAP-Flag-*SPT7 *were grown in YP broth containing 2% glucose. Lane 1 (ctrl) contains BY4741 transformed with the control vector YCp-TAP-Flag. TAP-Flag-Spt7 was purified through affinity chromatography on IgG-Agarose and eluted after cleavage with TEV protease. Approximately equal amounts of Spt7 were separated by 5% SDS-PAGE and Western blotted with anti-Flag antibody. The mobility of 250 and 150 kDa molecular mass markers are indicated on the left. Lane 3 contains one half of the wild-type sample applied in lane 2 to facilitate quantitation.

To determine if the components of the SAGA or SLIK complex influence the truncation of Spt7, we introduced *YCpDed-TAP-Flag-SPT7 *into yeast strain BY4741 (wild-type) and isogenic strains deleted for *GCN5*, *RTG2*, *SPT8, UBP8, ADA1 *and *SPT20*. Cells were grown in YPD media and protein extracts examined for Spt7 by Western blotting with anti-Flag antibody (Figure 2a). Two proteins with apparent molecular masses of approximately 220 and 200 kDa were present in wild-type cells containing *YCpDed-TAP-Flag-SPT7 *but not in control cells (compare lanes 1 and 2). The size of these molecules suggest that they represent the TAP-Flag tagged full-length (Spt7_SAGA_) and SLIK-forms (Spt7_SLIK_) of Spt7. The ratio of SAGA:SLIK forms of Spt7 was ~3:1 in the wild-type strain BY4741 (lane 2). This relative amount of Spt7_SLIK _was not reduced upon disruption of *GCN5*, *RTG2*, *ADA1, SPT20 *or *SPT8*. Since Spt20 and Ada1 are required for the integrity of the SAGA complex [6], the presence of Spt7_SLIK _in these deletion strains indicates that C-terminal processing can occur in the absence of the fully intact and functional complex. (We do note that there was an apparent decrease in the ratio of SAGA:SLIK forms in the *spt20Δ0 *strain and speculate that this may be due to differential stability of the forms in this strain background.) In addition, the wild-type level of Spt7_SLIK _seen in the *spt8 *deletion background suggests that the Spt8 interaction with Spt7 does not alter access of the putative processing enzyme(s) to the cleavage site. Furthermore, the lack of a requirement for Rtg2 indicates that the SLIK complex need not fully assemble to allow truncation of Spt7.

**Figure 3:**
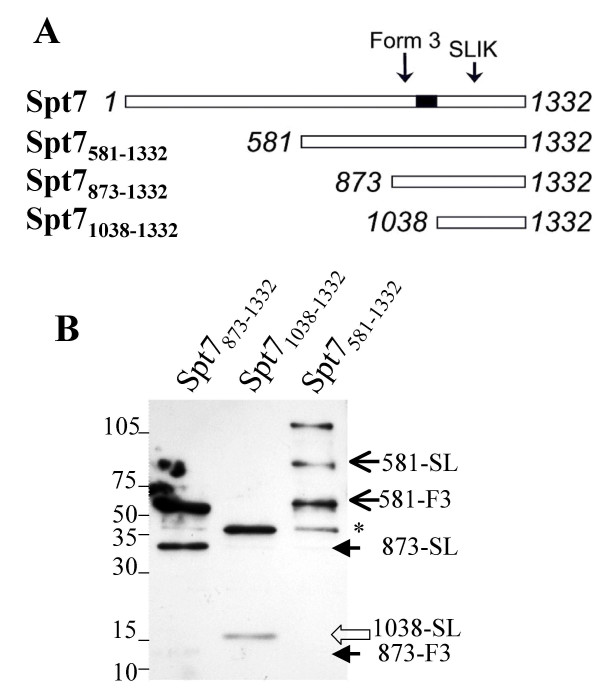
*Formation of truncated forms of Spt7 in N-terminally deleted derivatives*. **A**. N-terminally deleted derivatives of Spt7 were constructed containing amino acids 581–1332 (Spt7_581–1332_), 873–1332 (Spt7_873–1332_) and 1038–1332 (Spt7_1038–1332_). Note that each molecule is N-terminally TAP and Flag tagged to allow purification then detection of processed fragments. The position of the histone fold is shown with a black box and the approximate position of the Spt7_SLIK _and Spt7_Form3 _processing sites by arrows. **B**. *YCpDed-TAP-Flag*- Spt7_581–1332_, Spt7_873–1332 _and Spt7_1038–1332 _were expressed in BY3218 (*spt7Δ0*), tandem affinity purified and Spt7 forms detected by Western blotting with anti-Flag antibody after electrophoresis on an 8–15% gradient gel. Mobility of protein standards with the indicated molecular masses (kDa) is indicated on the left. Arrows on the right marked with 581, 873 and 1038 represent the mobility of truncated TAP-Flag- Spt7_581–1332_, Spt7_873–1332 _and Spt7_1038–1332_, respectively. * marks an additional cleavage product seen with Spt7_581–1332_.

Although still apparent, the amount of Spt7_SLIK _was reduced approximately three-fold in the strain deleted for the ubiquitin protease Ubp8 (Figure 2a, lane 6). We note that the *ubp8Δ0 *effect was found to vary from approximately two-fold to five-fold between different experiments (not shown). To determine whether the role of Ubp8 was the result of its involvement with SAGA, we examined the Spt7 forms in a strain deleted for Sgf11, a subunit of SAGA required for the association of Ubp8 with components of the complex [40-42]. As shown in Figure 2b, deletion of *sgf11 *partially reduced the level of Spt7_SLIK_. This effect was not to the same extent as deletion of Ubp8, perhaps because low levels of Ubp8 remain associated with SAGA in the absence of Sgf11 [40]. Nonetheless, while not eliminating other mechanisms, the result is consistent with a model whereby binding of Ubp8 to SAGA may alter the accessibility of the C-terminus of Spt7 to cleavage. Interestingly, another Flag-antibody reactive band, Spt7_Form3_, was evident in the sample lanes but not in the control. Since Spt7_Form3 _was somewhat obscured by a Flag antibody cross-reactive band, we analyzed the Spt7 forms in different strain backgrounds after first purifying TAP-Flag-Spt7 on IgG-agarose (Figure 2c). After purification a Flag-reactive molecule with an apparent mass approximately 20 kDa less than Spt7_SLIK _(also see Figure 3b) was revealed. The observed difference in mass of this molecule relative to intact Spt7 estimates an end-point at approximately amino acid residue 950. As was the case for Spt7_SLIK_, the level of this third form of Spt7 was comparable in each of the deletion strains (*gcn5Δ0*, *spt8Δ0*, *spt20Δ0*, *ada1Δ0*, *rtg2Δ0*) with the exception of *ubp8Δ0 *where it was reduced.

### C-terminal processing of Spt7 occurs in the absence of its N-terminal amino acid residues

The finding that Spt7_SLIK _and Spt7_Form3 _were formed in the absence of components of SAGA, including Spt20 and Ada1, suggested that processing is not linked directly to the activity of the complex. If processing were part of the functional dynamics of SAGA, that is, occurring as a result of its activities in the regulation of transcription, then one would predict that it would require Spt7 in a functional form. We thus examined whether N-terminal deletion derivatives of Spt7, expressed in BY3218 (*spt7Δ0*) would be processed (Figure 3). Each molecule was N-terminally TAP and Flag tagged to allow purification and identification of Spt7 forms. The two longest derivatives (residues 580–1332 and 873–1332) contain the elements of the protein required for partial function [6]. By comparison, the shortest derivative (residues 1038–1332) lacks the histone fold, which is required for interaction with other components of the complex [43]. A protein reduced by approximately 20 kDa, an estimated mass consistent with a truncation at the Spt7_SLIK _processing site, was seen for each of the derivatives (denoted as SL). A further truncation, approximately 20 kDa smaller than Spt7_SLIK _and potentially corresponding to processing at the Spt7_Form3 _site (F3), was evident for Spt7_581–1332 _and Spt7_873–1332 _(though at a reduced level). Note that the third-form equivalent was not seen for Spt7_1037–1332_, because its putative cleavage site is removed from this construct. This result suggests that the elements required for processing are intrinsic to the C-terminal end of Spt7 and that the N-terminus of the protein is not required. Furthermore, the appearance of a cleavage product for Spt7_1037–1332 _suggests that processing can take place independently from SAGA and is thus not linked to the action of the complex. For Spt7_581–1332_, a fourth form was apparent just below Form 3 (marked by *). We have not characterized this form further and can not exclude that it is a degradation product.

**Figure 4:**
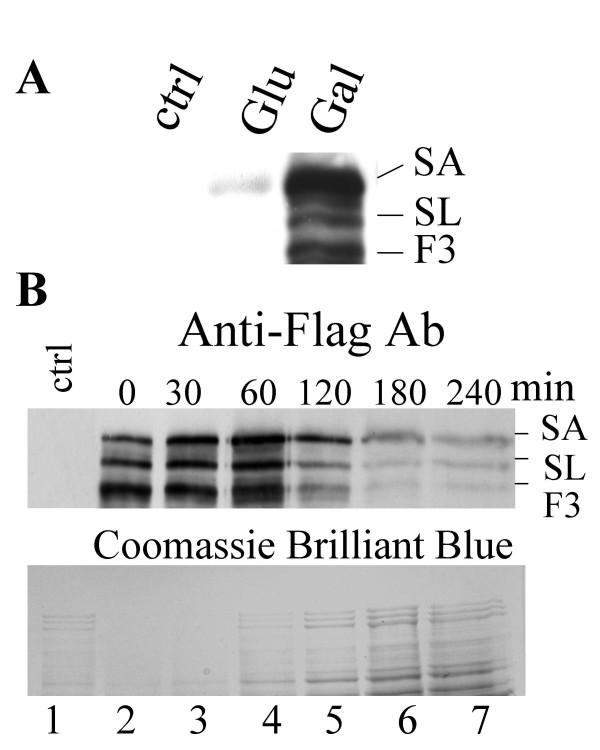
*Time course of the induction and decay of Spt7 forms*. **A**. CY1811 containing *YIplac211-GAL-Flag*_3_*-SPT7 *was grown in minimal media containing 2% raffinose then diluted 1:5 into YP media containing 2% glucose (lane 2) or 2%galactose (lane 3) and grown for 30 minutes. Extracts were prepared by glass bead disruption and 100 μg separated by SDS-PAGE (5%). Flag_3_-Spt7 was detected by Western blotting with anti-Flag antibody. Lane one contains 100 μg of protein extract prepared after growth of BY4741 in galactose-containing media. **B**. CY1811 containing *YIplac211-GAL – Flag*_3_*-SPT7 *was grown in raffinose. Expression of Spt7 was induced by the addition of galactose then inhibited by the addition of YP media containing 2% glucose. Cells were grown for 90 minutes which was empirically determined as time 0. Additional equal volume samples were taken at time 0, 30, 60, 120 and 240 minutes (lanes 2–7). Protein extracts were prepared and equal volumes separated by SDS-Page (5%). Flag_3_-Spt7 was detected by Western blotting (top panel) or stained with Coomassie Brilliant Blue (bottom panel).

If the forms of Spt7 were created progressively from one another in response to the activity of the complex, one would predict that the longer forms would be chased into shorter forms. We analyzed the appearance and disappearance of the Spt7 forms upon inducing expression of a triple Flag-tagged Spt7 under control of the *GAL10 *promoter (*GAL*-Flag_3_-Spt7; see Figure 1). As shown in Figure 4a, cells containing *GAL*-Flag_3_-Spt7 rapidly induce the three Spt7 forms when shifted from raffinose to galactose containing media but not upon shifting to glucose containing media. Figure 4b is a time course of the appearance and disappearance of the Spt7 forms after induction in galactose containing media and subsequent shift to media containing glucose. Expression of the forms continued to increase after promoter shut-off until approximately the 60 minute time point. All three forms increased at approximately the same rate. After 60 minutes, the amounts of the Spt7 forms began to decay. This was in contrast to total protein, which continued to increase (comparing the Western blot versus the stained gel in Figure 4b). Similar to their appearance, the three forms disappeared at approximately the same rate. The longer forms were not chased into the shorter molecules, suggesting that Spt7_SLIK _and Spt7_Form3 _are produced early after the synthesis of Spt7_SAGA _and not sequentially as the result of the action of the complex. Combined with the results above, processing appears inherent to the C-terminus of the molecule and occurs independently from the action of the SAGA complex.

### Further characterization of the 160 kDa form of Spt7

**Figure 5:**
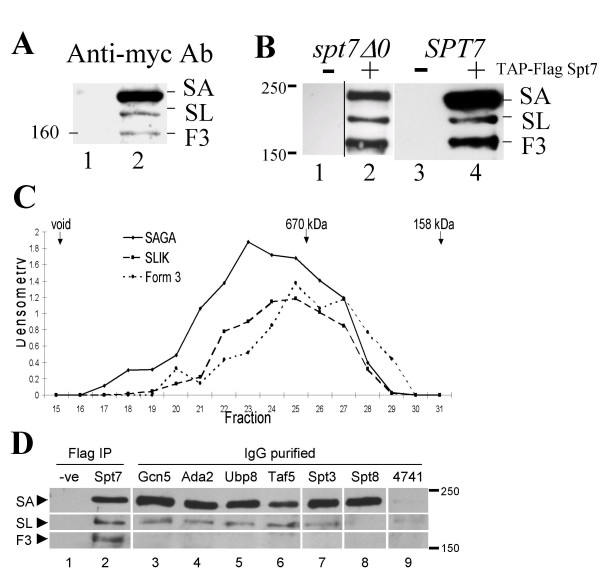
*Characterization of Spt7 Form3*. **A**. YCp88-myc was transformed into BY4741 (lane 1, ctrl) and YCp88-myc-*SPT7 *into BY4741 (lane 2). Crude protein extracts were prepared and Western blotted with anti-myc antibody. The mobility of a 160 kDa molecular weight marker is indicated. Spt7_Form3 _is marked as F3. **B**. BY4741 (wild-type, right panel) and BY3218 (*spt7Δ0*, left panel) were transformed with YCpDed-TAP-Flag (lanes 1 and 3) or YCpDed-TAP-Flag-*SPT7 *into (lanes 2 and 4). Extracts were prepared by grinding 1 litre of cells in liquid nitrogen, and subjected to TAP purification. 20 μl of the peak fractions were blotted with anti-Flag antibody. **C**. *Gel filtration chromatography of Spt7 from crude yeast extracts*. 8.5 mg of protein extract was prepared from yeast strain BY4741 containing YCpDed-TAP-Flag-*SPT7 *and applied to a Superose 6 HR10/30 column (Pharmacia). 500 μl fractions were collected, precipitated with 10% trichloroacetic acid, separated on a 5% SDS-polyacrylamide gel and Western blotted with anti-Flag antibody. Relative amounts of each of the three forms as determined by densitometry are shown as are the peak fraction for elution of the indicated molecular mass standards. **D**. Wild-type (4741; lane 9) or strains containing C-terminally TAP-tagged Gcn5, Ada2, Ubp8, Taf5, Spt3 and Spt8, expressing Flag-Spt7 (lanes 3–8) were grown in YP media containing 2% glucose to an OD_600 _= 2.0. Twenty-five milligrams of crude lysate was purified on IgG agarose. Bound protein was separated by 5% SDS-PAGE and Flag-Spt7 forms detected with anti-Flag antibody. To demonstrate the position of the three Spt7 forms lanes 1 and 2 are extracts of BY4741 transformed with YCpDed-Flag-*SPT7 *that have been purified on Sepharose 4B (-'ve) or anti-Flag antibody coupled to Sepharose (Spt7) then Western blotted with anti-Flag antibody. The positions of molecular mass markers, Spt7_SAGA _(SA), SPT7_SLIK _(SL) and Spt7_Form3 _(F3) are indicated.

To eliminate the possibility that the appearance of Spt7_Form3 _was related to blotting with Flag antibody or to the TAP-tag, we expressed N-terminally myc-tagged Spt7, separated crude protein extract by SDS-PAGE and blotted with anti-myc antibody (Figure 5a). A band approximately 20 kDa smaller than myc-Spt7_SAGA_, likely myc-Spt7_SLIK _was present in wild-type cells containing *YCp88-myc-SPT7 *but not in control cells (compare lanes 1 and 2) as was the third-form at an apparent mass of ~160 kDa. To determine if Spt7_Form3 _was an artifact of the partial TAP purification or expression in cells also containing a wild-type copy of *SPT7*, we purified TAP-Flag-Spt7 from BY4741 (wild-type) and BY3281 (*spt7Δ0*) through both tandem affinity steps, IgG-Agarose and Calmodulin-Sepharose. As shown in Figure 5b, a ~160 kDa, Flag-detected protein was apparent after purification (lanes 2 and 4) and not in a mock purification (lanes 1 and 3) in both strain backgrounds.

To examine if Spt7_Form3 _is found within complexes, TAP-Flag-Spt7 was expressed in BY4741 and a crude protein extract applied to a Superose 6 HR10/30 gel filtration column. The elution profile of the different forms of Spt7 was examined by Western blotting with anti-Flag antibody and the relative amounts of the three forms of Spt7 determined by densitometry (Figure 5c). All three forms of Spt7 eluted in fractions corresponding to a high molecular mass, the largest being Spt7_SAGA_, followed by Spt7_SLIK_. Spt7_Form3 _eluted from the column with an average apparent molecular size slightly less than Spt7_SLIK_. To determine if Spt7_Form3 _was associated with components of the SAGA complex, extracts were prepared from strains containing N-terminally Flag-tagged Spt7 and TAP-tagged versions of Gcn5, Ada2, Ubp8, Taf5, Spt3 and Spt8. Complexes containing the TAP-tagged molecules were partially purified and the presence of the different forms of Spt7 analyzed by Western blotting with anti-Flag antibody (Figure 5d, lanes 3–9). Identification of the forms was established by immunoprecipitation of Flag-Spt7 from crude extracts with anti-Flag antibody (compare lanes 1 and 2). As expected from the interaction of Spt8 with the C-terminal end of Spt7 [4,6], Spt8 interacted only with full-length Spt7 (lane 8). The other SAGA components interacted with both Spt7_SAGA _and Spt7_SLIK_. Spt7_Form3 _did not co-purify with the SAGA components to the extent of either Spt7_SAGA _or Spt7_SLIK_.

The function of Spt7_Form3 _is unknown. The fact that Spt7_Form3 _appears as stable as the longer forms is consistent with it having a function(s), likely distinct from components of the SAGA complex. It is interesting to note that Spt7 has been found to associate with Ccl1, Rtf1 and Prp43, and that these interactions are not reciprocated by C-terminally TAP-tagged Spt7 or generally by other components of the SAGA complex (*Saccharomyces Genome Database*). In addition, we previously observed a possible SAGA-independent form of Spt7 by fractionation of crude extracts on a Mono Q column [27]. We also must consider an alternative model, whereby Spt7_Form3 _is an intermediate along a pathway of degradation. In this regard, we did observe an apparent increase in Spt7_Form3 _during TAP-purification.

## Conclusion

In this report we show that processing of the C-terminus of Spt7 to the form found in the SLIK complex and to a novel third form does not require a functional SAGA complex. C-terminal truncation occurs in the absence of key components of SAGA and SLIK complexes. Spt20 and Ada1 are necessary for the integrity of a complete SAGA complex with their deletion resulting in severe transcriptional effects [6,22], yet processing occurs in their absence. In addition, since the truncated Spt7 molecules were present in a *gcn5Δ0 *strain, histone acetylation is not required for processing. Also suggesting that processing of Spt7 is independent of the function of SAGA, we found that a truncated derivative of Spt7 that lacks the histone fold is capable of being processed in a strain lacking full-length Spt7. Finally a time course revealing the appearance and disappearance of the Spt7 forms showed that Spt7_SAGA _is not chased into the shorter forms. We thus suggest that processing of Spt7 is an independent event in the biosynthesis of distinct Spt7 containing complexes rather than being linked to transcriptional regulation by the SAGA complex.

Processing of Spt7 showed partial dependence on the ubiquitin protease Ubp8. The role of Ubp8 is unclear but based on a partial effect upon deletion of Sgf11, it may require the association of Ubp8 with SAGA. We were intrigued by the possibility that the effect of Ubp8 may be related to our previous observation of the ubiquitylation of Spt7 dependent on the E3-ubiquitin ligase Tom1 [44]; however, because the relative amounts of the Spt7 forms appear unchanged in a *tom1Δ0 *strain (not shown) we have concluded that these observations are unrelated.

Our study has identified a third form of Spt7 with an apparent molecular mass of ~160 kDa. The identification of this form with three different tagged derivatives suggests that it is not an artifact of tagging; however, we can not fully exclude the possibility that N-terminal tagging results in its appearance. Gel-filtration experiments suggest that Spt7_Form3 _is found within a complex; albeit, distinct from SAGA or SLIK. As Spt7 was N-terminally tagged, the mobility of Spt7_Form3 _on SDS-PAGE suggests a molecule containing amino acid residues 1 to ~950. Determining the size of the released C-terminal fragment would facilitate mapping of the cleavage site; however, we have been unable to identify this molecule using C-terminally TAP-tagged Spt7.

## Methods

### Yeast strains

The wild-type yeast strains BY4741 (*MATa hisΔ0 leu2Δ0 met1Δ0 ura3Δ0*) and BY4742 (*MATα hisΔ0 leu2Δ0 lys2Δ0 ura3Δ0*) and the consortium constructed isogenic knockout strains [45] BY3218 (*spt7Δ0*), BY10809 (*ubp8Δ0*), BY17285 (*gcn5Δ0*), BY14619 (*rtg2Δ0*), BY12666 (*spt8Δ0*), BY14433 (*sgf73Δ0*), BY13418 (*sgf29Δ0*), BY2781 (*sgf11Δ0*), BY1038 (*ada1Δ0*) and BY17390 (*spt20Δ0*), and strains containing C-terminally TAP-tagged SAGA components were purchased from Open Biosystems or Research Genetics. CY1811 is a derivative of BY4741 that contains a *YIplac211-GAL-Flag*_3_*-SPT7 *integrated at the endogenous locus.

### DNA constructs

*YCpDed-TAP *is a derivative of the *LEU2 *containing centromeric plasmid YCplac111 [46] that contains a *DED1 *promoter [9] driving expression of a TAP epitope. The *DED1 *promoter was synthesized by PCR using oligonucleotides 4149-1 and 4149-2 (see Table 1) and cloned as a *Pst*I-*Bam*HI fragment into YCplac111. The TAP epitope was subsequently cloned as a *Bam*HI to *Sst*I fragment into this molecule after its PCR using oligonucleotides 4149-4 and 4168-1 and pFA6a as the template (kindly provided by Kathy Gould). The coding sequence for the Flag-epitope was cloned into this molecule at the engineered *Not*I site using oligonucleotides 4213-1 and 4213-2 to give YCpDed-TAP-Flag. Epitope-tagged Spt7 was constructed through PCR amplification of two tandem parts of the 3999 base pair gene using oligonucleotides 4051-1 and 4051-2 for the 5'-segment and 4009-1 and 4009-2 for the 3'-segment, respectively. The two segments were ligated using the common internal *Sal*I restriction site and cloned into YCpDed-TAP-Flag as a *Not*I-*Sst*I fragment to generate YCpDed-TAP-Flag-*SPT7*. A molecule expressing Myc-tagged Spt7 was constructed by cloning a *Not*I-*Eco*RI fragment from YCpDed-Tap-Flag-*SPT7 *into the *URA3 *centromeric plasmid YCp88-myc [9] to generate YCp88-myc-*SPT7*. Single Flag-tagged Spt7 similarly contains a Met-Tyr-Lys-Asp_4_-Lys coding sequence inserted directly before the *Not*I site.

**Table 1:** Oligonucleotides used in this study

**Oligo#**	**Sequence (5'-3')**	**Construct(s)**
4009-1	GCGGCCGCTGGAAGAAAAGGATTGAATATGG	3' fragment of *SPT7*
4009-2	GAATTCTATTCAACTATTTAGCGCGCTC	
4051-1	GCGGCCGCAATGACTGAAAGAATACCAATAAAG	5'fragment of *SPT7*
5051-2	CCAAATCGTCTCTATCGTCATCC	
4536-1	GATGCGGCCGCGATGGAAGACGCTTCCGTG	5' truncations of *SPT7*
4536-2	GATCGGCCGCCGGTATCAACAGGCCAGAC	
4213-1	GGCCGACTACAAGGACGACGATGACAAGGC	N-terminal Flag epitope
4213-2	GGCCGCCTTGTCATCGTCGTCCTTGTAGTC	
4149-4	GAGCTCGCGGCCGCATAATCAAGTGCCCCGGAG	TAP-tag
4168-1	GGATCCATGAAAGCTGATGCGCAACAAATT	
4356-1	GATGCGGCCGCGATGGAAGACGCTTCGTG	*DED1 *promoter
4356-2	GATGCGGCCGCCGGTATCAACAGGCCAGAC	
2764-1	AACTGCAGTAATACGCTTAACTGCTC	*GAL10 *promoter
2764-2	CCCAAGCTTGACGTTAAAGTATAGAGGT	
2821-1	ACGAAGCTTACCATGGACTACAAGGACGACGAT	Met-Flag tag
2821-2	TTGAGCTCTGCGGCCGCCTTGTCATCGTCGTCC	

TAP-tagged derivatives of Spt7 with 752, 460 and 295 C-terminal amino acids were constructed by PCR using oligonucleotides 4009-1, 4536-1 and 4536-2 as 5' primer, and 4009-2 as 3' primer. Each was cloned into YCpDed-TAP-Flag as a *Not*I-*Eco*RI fragment to generate YCpDed-TAP-Flag-*SPT7*_580–1332_, YCpDed-TAP-Flag-*SPT7*_872–1332 _and YCpDed-TAP-Flag-*SPT7*_1038–1332_.

The parent construct for plasmids expressing Flag_3_-Spt7 is pCB1450. This molecule contains a N-terminal Flag epitope (engineered with oligonucleotides 2821-1 and 2821-2) flanked upstream by the *Pst*I site of the pUC polylinker and downstream by a *Not*I site. Genes to be expressed can be inserted as *Not*I-(*Sac*I)*Eco*RI fragments. The polylinker *Hin*dIII site was deleted allowing insertion of promoter fragments as *Pst*I-*Hin*dIII fragments. The *GAL10 *promoter was synthesized by PCR using oligonucleotides 2764-1 and 2764-2 and inserted into pCB1450. *SPT7 *from YCpDed-TAP-Flag-*SPT7 *was inserted as a *Not*I-*Eco*RI fragment and two additional Flag epitopes were inserted at the *Not*I site using oligonucleotides 4213-1 and 4213-2. For integration into yeast the *GAL-Flag*_3_*-SPT7 *module was inserted into YIplac211 [46]. Integration into the endogenous locus was possible after digestion with *Sal*I.

### TAP purification

Tandem affinity purification (TAP) was carried out as described [47] with minor modifications. Yeast cells were grown in YPD to an A_600 _= 1.5 and lysed by grinding in liquid nitrogen [48]. All further steps were performed at 4°C. Per liter of starting culture, cell extracts were suspended in ~5 ml of IPP150 (10 mM Tris-HCl (pH 8.0), 150 mM NaCl, 0.1% NP-40) with protease inhibitors (1 mM PMSF, 0.1 mM benzamidine hydrochloride, 2 μg/ml pepstatin A, 2 μg/ml leupeptin and 0.1 mg/ml trypsin inhibitor). The extract was cleared by centrifugation at 40,000 g for 1 h and incubated with 400 μl of IgG-Agarose (Sigma-Aldrich Canada Ltd) suspension for 2 h. After washing with 30 ml of IPP150 and 10 ml of TEV cleavage buffer (10 mM Tris-HCl (pH 8.0), 150 mM NaCl, 0.5 mM EDTA, 1 mM dithiothreitol, 0.1% NP-40), IgG beads were suspended in 1 ml of TEV cleavage buffer and incubated with ~100 U TEV protease for 2 h. 3 μl of 1 M CaCl_2 _was added to the TEV-cleaved eluent which was then diluted with 3 ml of calmodulin binding buffer (10 mM Tris-HCl (pH 8.0), 150 mM NaCl, 1 mM magnesium acetate, 1 mM imidazole, 2 mM CaCl_2_, 1 mM beta-mercaptoethanol, 0.1% NP-40). For the second step of purification the protein was applied to 400 μl of Calmodulin-Sepharose (Stratagene Inc) and incubated for 2 h. After the calmodulin beads were washed with 30 ml of calmodulin binding buffer, bound protein was eluted with calmodulin elution buffer (10 mM Tris-HCl (pH 8.0), 150 mM NaCl, 1 mM magnesium acetate, 1 mM imidazole, 20 mM EGTA, 1 mM beta-mercaptoethanol, 0.1% NP-40), in 200 μl fractions. 20 μl of the peak fractions were separated by 5% SDS-PAGE and subjected to Western blotting.

### Western blotting

Yeast extracts were prepared by glass bead disruption [9] or grinding in liquid nitrogen [48]. Protein was separated by SDS polyacrylamide gel electrophoresis, transferred to PVDF membranes (Roche Applied Science) using a wet transfer system (Bio-Rad) in 48 mM Tris, 40 mM glycine, 0.0375% (6.5 mM) SDS and 20% methanol for 1 h at 100 V and detected using a 20% solution of Immobilon Western (Millipore Corp). Anti-Flag antibodies (M2, Sigma-Aldrich Canada Ltd) were used at a ratio of 1:4000; anti-myc antibody (Myc1-9E10 cell line) was used at a ratio of 1:2000. Densitometric scanning of films was performed using AlphaImager 3400 software (Alpha Innotech, Inc). Serial dilutions of samples were analyzed to provide standard curves for suitable film exposure.

### Time course of the induction and decay of Spt7

A 10 ml culture of CY1811 was grown to an A_600 _~3.0 in minimal media containing 0.6% casamino acids (Difco) and 2% raffinose. The culture was diluted with 10 ml of prewarmed media containing 2% galactose and grown for 30 minutes at 30°C. 80 ml of YP media containing 2% glucose was added and the cells grown for 90 minutes. This became time 0, and a 15 ml sample was taken. Cells were pelleted by centrifugation, washed in ice-cold water and frozen at -80°C. Additional samples were taken after 30, 60, 120 and 240 minutes. Yeast extracts were prepared by glass bead disruption in 20 mM Hepes-NaOH (pH 8.0), 240 mM NaCl containing protease inhibitors. Equal volumes were separated by SDS-PAGE (5%) and Western blotted with anti-Flag antibody.

### Gel-filtration chromatography

Approximately 8.5 mg of whole cell extract from BY4741 containing YCpDed-TAP-Flag-*SPT7 *was prepared in 40 mM Tris-HCl (pH 7.7), 300 mM NaCl, 0.1% Nonidet P-40 and 10% glycerol, and subsequently applied to a Superose 6 HR10/30 column (Pharmacia Inc) at a flow rate of 0.3 ml/min. Protein from 250 μl aliquots of 500-μl selected fractions were precipitated with 10% trichloroacetic acid, acetone washed, resuspended in SDS loading buffer and separated on a 5% SDS-polyacrylamide gel.

## Authors' contributions

SH carried out the analysis of Spt7 in yeast deletion strains, copurifications and mapping studies. GL performed analyses with Spt7 deletion constructs and biochemical fractionation of Spt7. AM performed functional analyses of Spt7 alleles. JG carried out the time course analysis of Spt7 synthesis and degradation. CB directed the experiments and wrote the manuscript.
